# Successful Vaginal Delivery despite a Huge Ovarian Mucinous Cystadenoma Complicating Pregnancy: A Case Report

**Published:** 2013-12

**Authors:** Dipak Mandi, Ratan Chandra Mondal, Debasish Bhar, Ashok Kumar Maity, Malay Kr. Nandi, Kinkar Singh

**Affiliations:** 1 Department of Obstetrics and Gynecology, Midnapore Medical College and Hospital, Paschim Midnapore, West Bengal, India;; 2 Department of Anesthesiology, Midnapore Medical College and Hospital, Paschim Midnapore, West Bengal, India;; 3 Department of Pathology, Midnapore Medical College and Hospital, Paschim Midnapore, West Bengal, India

**Keywords:** Mucinous cystadenoma, Pregnancy, Intrauterine growth retardation

## Abstract

A 22-year-old patient with 9 months of amenorrhea and a huge abdominal swelling was admitted to our institution with an ultrasonography report of a multiloculated cystic space-occupying lesion, almost taking up the whole abdomen (probably of ovarian origin), along with a single live intrauterine fetus. She delivered vaginally a boy baby within 4 hours of admission without any maternal complication, but the baby had features of intrauterine growth restriction along with low birth weight. On the 8^th^ postpartum day, the multiloculated cystic mass, which arose from the right ovary and weighed about 11 kg, was removed via laparotomy. A mucinous cystadenoma with no malignant cells in peritoneal washing was detected in histopathology examination. This report describes a rare case of a successful vaginal delivery despite a huge cystadenoma of the right ovary complicating the pregnancy.

## Introduction

Mucinous cystadenomas are benign epithelial ovarian tumors which tend to be unilateral and multilocular with smooth surface and contain mucinous fluid. They comprise 12%-15% of all ovarian tumors. Seventy-five percent of all mucinous tumors are benign, while 10% are borderline and 15% are invasive carcinomas. Benign mucinous tumors are most common in the third to fifth decades of life and may be 20-30 cm in size.^1^ The incidence of ovarian cysts during pregnancy is less than 5%, and most of them are benign in nature. Giant cysts are found in less than 1% of the cases of ovarian cysts with pregnancy.^2^ Torsion is the most common and serious complication of benign ovarian cysts during pregnancy, particularly in the first trimester. The cyst may rupture in the peritoneal cavity due to torsion.^3^ Rupture of ovarian cysts may also occur during labor, delivery, immediate postpartum period, and surgery. Several cases of giant ovarian mucinous cystadenomas complicating pregnancy^2^^,^^4^^,^^5^ have been reported. In the reported cases, however, either the cysts were removed during pregnancy or the baby was delivered via Caesarean section along with the removal of the cyst. In our case, pregnancy was complicated by a huge right ovarian mucinous cystadenoma, which resulted in intrauterine fetal growth restriction (IUGR). Be that as it may, the mother had an uncomplicated vaginal delivery. 

## Case Description

A 22-year-old mother (gravida 2, para 1, with about 37 weeks of amenorrhea) was admitted through emergency to the Labor Room of Midnapore Medical College and Hospital, Midnapore, India, with chief complaints of intermittent lower abdominal pain and watery vaginal discharge since the previous evening. She had been married for 9 years and had a girl baby via institutional vaginal delivery 7 years previously. The patient was from a poor socioeconomic status and was referred from the local Block Primary Health Center (BPHC) as a case of pregnancy with a huge ovarian cyst. 

The medical records available from the patient showed that she had been previously admitted to our institution for a disproportionate increase in the abdominal size in the second trimester of pregnancy. Ultrasonography detected a single live intrauterine fetus of 22 weeks of gestation, together with a huge cystic mass arising from the right adnexa. At the time, she refused surgical intervention and returned home against medical advice. After 4 weeks, repeated ultrasonography also revealed a huge multiloculated cystic space-occupying lesion, almost taking up the entire abdomen (ovarian origin), along with a single live intrauterine fetus of 26 weeks of gestation (maturity grade 3 with adequate liquor). Furthermore, the placenta was adhered to the posterior upper segment. When the patient was admitted to our institution with abdominal pain, the gestation period was calculated to be about 37 weeks based on previous ultrasonography reports. 

General survey and systemic examination showed no abnormality, except for mild pallor and poor nutritional status. On abdominal examination, no fetal parts were palpable due to huge tense abdominal swelling. Even the fetal heart sound could not be located, although the patient perceived fetal movement. Internal examination on admission revealed 6 cm cervical dilatation with 90% effacement, vertex presentation, station +1, and bulged membrane. She delivered vaginally a boy baby within 4 hours of admission. The baby had a good Apgar score at 1 minute and 5 minutes, but his birth weight was 1.75 kg. A pediatrician was consulted on account of the baby’s low birth weight and features of IUGR. Labor and postpartum period were uneventful. 

**Figure 1:**
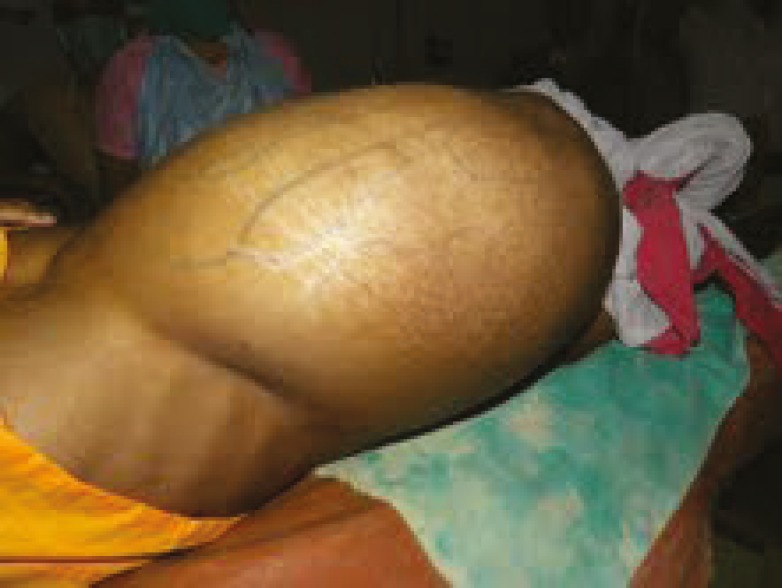
The patient’s abdominal swelling after vaginal delivery is demonstrated here

**Figure 2:**
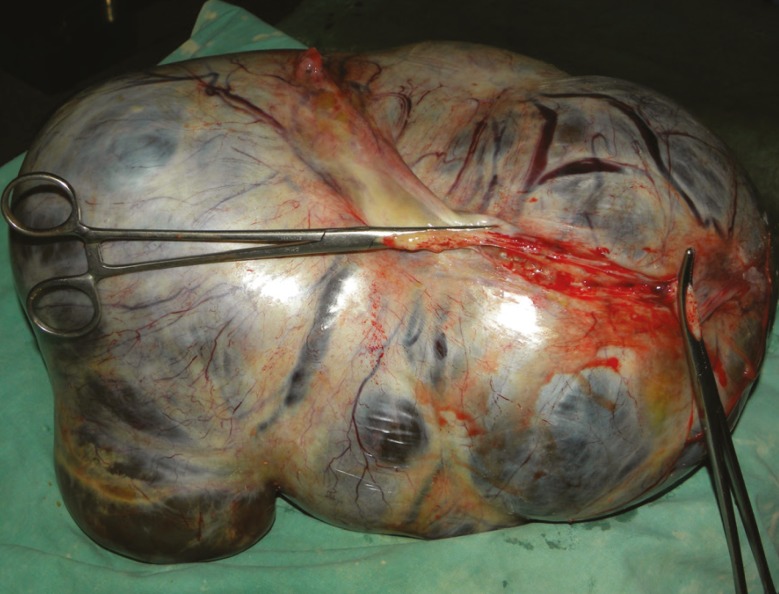
This cystic mass (approximately 40 cm×30 cm×25 cm in size, pinkish in color, and with a smooth surface) arising from the right ovary was removed via laparotomy

Given the patient’s history, clinical examination, and previous sonography reports, the abdominal mass was provisionally diagnosed as cystic adnexal swelling. She had a huge abdominal swelling even after the delivery of the baby. Figure 1 demonstrates the patient’s hugely distended abdomen after vaginal delivery. After proper counseling, decision for laparotomy was taken in the postpartum period. All the preoperative investigations were within normal limits. On the 8^th^ postpartum day, laparotomy was performed under general anesthesia. A cystic mass (approximately 40 cm×30 cm×25 cm in size, pinkish in color, and with a smooth surface) arising from the right ovary was found. The left fallopian tube and ovary was healthy. The right ovary, along with the mass and fallopian tube, was removed. Infracolic omentectomy and left tubectomy was done (as per patient and her husband’s consent). The intact cyst was multiloculated, weighed 11,000 gm, and was filled with fluid. Figure 2 demonstrates the cystic mass after it was removed via laparotomy. There was mild omental adhesion, but no ascites was observed. Specimens and peritoneal washing were sent for histopathology examination. The intraoperative and postoperative periods were uneventful, and she was discharged on the 8^th^ postoperative day. In subsequent follow-up, no abnormality was detected.

**Figure 3:**
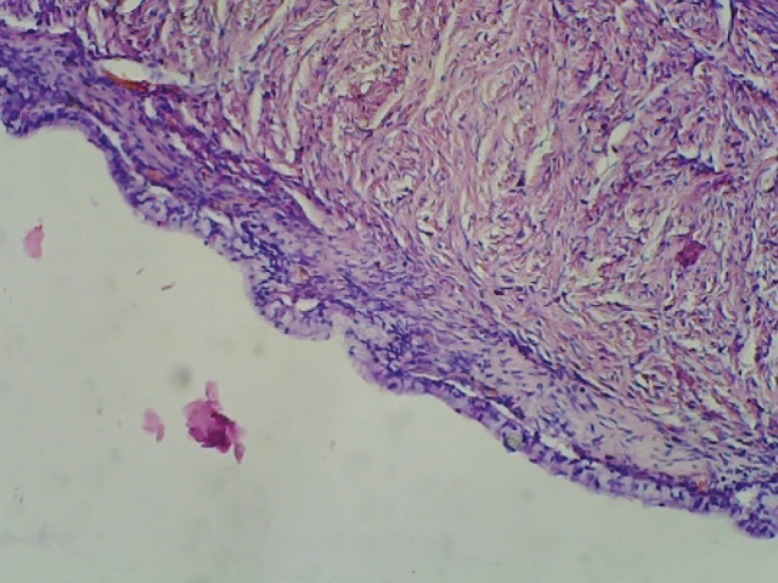
Histopathology of the mass shows features of a mucinous cyst adenoma

The ovarian cyst was sectioned in the Pathology Department of our institution. The inner surface of the cyst exhibited multiple trabeculae, without any solid component or hemorrhagic area. The cyst was filled with mucinous fluid. Unfortunately, the pathologist failed to provide us with the photograph of the macroscopic cut section. The mucinous ovarian cyst had features of infarction, so the histopathology slides could not be stained with specific Periodic Acid Schiff (PAS) stain to confirm diagnosis with an alternative method. The histopathology examination report revealed features of a mucinous cystadenoma. Figure 3 shows the histopathology of the mass, demonstrating features of a mucinous cystadenoma with no malignant cells in peritoneal washing.

## Discussion

The most common benign adnexal masses during pregnancy are cystic teratomas (36%), followed by cystadenomas (15%).^2^ Several cases of ovarian mucinous cystadenomas in pregnancy have been reported in the literature.^2^^,^^4^^-^^8^ Yenicesu GI et al.^4^ and Qublan HS et al.^6^ both described removal of right ovarian mucinous cystadenomas weighing around 6 kg after Caesarean section. The cysts in both cases were very similar to that in our patient. In our case, however, the baby was delivered vaginally without any intrapartum and postpartum complications despite the presence of such a huge ovarian mass. This is indeed a rare case because there have hitherto been no reports in the existing literature on vaginal delivery complicated by such a huge ovarian mucinous cystadenoma. 

Noreen H et al.^5^ reported term vaginal delivery of a grand multipara (aged 30 years) after the removal of a huge left ovarian mass (42 cm×40 cm×20 cm) at 30 weeks of gestation. Balat O et al.^7^ also reported an unthreatened late pregnancy with a huge mucinous cystadenoma of the left ovary, diagnosed sonographically at 26 weeks of pregnancy. 

Any adnexal mass smaller than 5 cm in size during pregnancy rarely causes symptoms. Therefore, the size of the tumor as well as its ultrasound characteristics, color Doppler flow, and symptoms is important in determining the management of pregnant patients with adnexal masses. Symptomatic, solid, bilateral, and complex lesions should be subjected to surgery whenever discovered. Moreover, unilateral simple ovarian cysts, 5-8 cm in size, should be evaluated sonologically up to 16-18 weeks and if they fail to regress or if they increase in size, surgical intervention should be undertaken. It is also advisable that any surgical intervention in the first trimester be avoided if possible because of the high rate of spontaneous abortion. The optimum time for surgical intervention is 16-18 weeks of gestation. 

In our case, the patient was referred to us at 22 weeks of gestation, at which time ultrasonography revealed a huge adnexal cyst with a single live intrauterine fetus. The patient was advised and counseled for surgical intervention, but she refused to cooperate and left against medical advice.

Several cases of huge ovarian mucinous cystadenomas with features of maternal virilization and fetal growth restriction have been reported in the literature.^4^^,^^5^^,^^8^ In our case, the placenta had grade 3 maturity at 26 weeks of gestation; nonetheless, the details of fetal biometry and Doppler study were not available. Clinically, the baby had features of asymmetric IUGR. This may be either due to the prominent vascularity of the tumor, originating from the ovarian vessels, or due to the compressive effect of the tumor on the uterine blood supply. The mother, however, had no features of virilization. 

## Conclusion

With the increase in the use of ultrasound in the first trimester of pregnancy, the reported incidence of the ovarian cyst with pregnancy is also increasing. All ovarian cysts during pregnancy should be followed up sonographically to prevent their adverse effects on pregnancy. If the cysts fail to regress or if they increase in size, surgical intervention is required (preferably in the second trimester). Patients in whom an asymptomatic mass is noted at or near term may be considered for delivery via Caesarean section with thorough surgical evaluation of the adnexa.
